# Simulation platform: cloud-computing meets computational neuroscience

**DOI:** 10.1186/1471-2202-12-S1-P346

**Published:** 2011-07-18

**Authors:** Tadashi Yamazaki, Hidetoshi Ikeno, Yoshihiro Okumura, Shunji Satoh, Yoshimi Kamiyama, Yutaka Hirata, Keiichiro Inagaki, Akito Ishihara, Takayuki Kannon, Shiro Usui

**Affiliations:** 1 RIKEN BSI-TOYOTA Collaboration Center, RIKEN Brain Science Institute, Wako, Saitama, 351-0198, Japan; 2 School of Human Science and Environment, University of Hyogo, Himeji, Hyogo, 670-0092, Japan; 3 Neuroinformatics Japan Center, RIKEN Brain Science Institute, Wako, Saitama, 351-0198, Japan; 4 Graduate School of Information Systems, University of Electro-Communications, Chofu, Tokyo, 182-8585, Japan; 5 School of Information Science and Technology, Aichi Prefectural University, Nagakute, Aichi, 480-1198, Japan; 6 Faculty of Engineering, Chubu University, Kasugai, Aichi, 486-8501, Japan; 7 Laboratory for Neuroinformatics (Computational Science Research Program), RIKEN Brain Science Institute, Wako, Saitama, 351-0198, Japan; 8 School of Information Science and Technology, Chukyo University, Toyota, Aichi 470-0393, Japan; 9 Laboratory for Neuroinformatics, RIKEN Brain Science Institute, Wako, Saitama 351-0198, Japan

## 

Computational models and theoretical tools are essential components in computational neuroscience. A number of models and tools have been developed and registered at various online databases such as ModelDB and J-Node Platforms. Yet, the reuse of such resources still remains quite difficult. For example, to carry out a computer simulation of a model, we have to download the program from the database, extract, read instructions, compile if the program is written in a general programming language such as C, install the appropriate neural simulator if it is written for a simulator such as GENESIS, NEURON, and NEST, and finally we may be ready to do it, if no problems occurs during all the setup mentioned above. How can we avoid this hustle?

**Figure 1:**
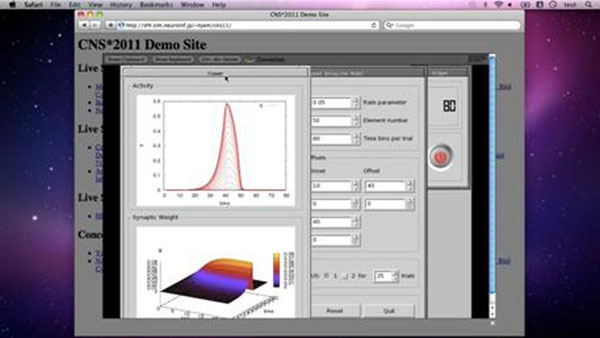
A screenshot of a web browser during a computer simulation.

As a solution of it, we introduce a cloud-based system for online computer simulation called Simulation Platform. Simulation Platform is a cloud of virtual machines running GNU/Linux. On a virtual machine, various software including developer tools such as compilers and libraries, popular neural simulators, and scientific software such as Gnuplot, R and Octave, are pre-installed. When a user posts a request, a virtual machine is assigned to the user, and the simulation starts on that machine. The user can remotely access the virtual machine through a web browser and carries out the simulation interactively (a screenshot is shown in Fig. 1). There is no need to install any software. It only requires a web browser. Therefore, Simulation Platform provides an ubiquitous computing environment for computational neuroscience research so as to free neuroscientists from tedious computer administration tasks and allow them to solely concentrate on their science. A demo site is open at http://sf4.sim.neuroinf.jp/~tyam/cns11/.

